# Inertial Sensing to Determine Movement Disorder Motion Present before and after Treatment

**DOI:** 10.3390/s120303512

**Published:** 2012-03-12

**Authors:** Wesley J. E. Teskey, Mohamed Elhabiby, Naser El-Sheimy

**Affiliations:** 1 Department of Geomatics Engineering, 2500 University Drive NW, University of Calgary, Calgary, Alberta T2N 1N4, Canada; E-Mails: mmelhabi@ucalgary.ca (M.E.); elsheimy@ucalgary.ca (N.E.S.); 2 Public Works Department, Faculty of Engineering, Ain Shams University, Khalifa El-Maamon st, Abbasiya sq, Cairo 11566, Egypt

**Keywords:** accelerometer, gyroscope, Parkinson’s disease, essential tremor

## Abstract

There has been a lot of interest in recent years in using inertial sensors (accelerometers and gyroscopes) to monitor movement disorder motion and monitor the efficacy of treatment options. Two of the most prominent movement disorders, which are under evaluation in this research paper, are essential tremor (ET) and Parkinson’s disease (PD). These movement disorders are first evaluated to show that ET and PD motion often depict more (tremor) motion content in the 3–12 Hz frequency band of interest than control data and that such tremor motion can be characterized using inertial sensors. As well, coherence analysis is used to compare between pairs of many of the six degrees-of-freedom of motions under evaluation, to determine the similarity in tremor motion for the various degrees-of-freedom at different frequency bands of interest. It was quite surprising that this coherence analysis depicts that there is a statistically significant relationship using coherence analysis when differentiating between control and effectively medicated PD motion. The statistical analysis uncovers the novel finding that PD medication induced dyskinesia is depicted within coherence data from inertial signals. Dyskinesia is involuntary motion or the absence of intended motion, and it is a common side effect among medicated PD patients. The results show that inertial sensors can be used to differentiate between effectively medicated PD motion and control motion; such a differentiation can often be difficult to perform with the human eye because effectively medicated PD patients tend to not produce much tremor. As well, the finding that PD motion, when well medicated, does still differ significantly from control motion allows for researchers to quantify potential deficiencies in the use of medication. By using inertial sensors to spot such deficiencies, as outlined in this research paper, it is hoped that medications with even a larger degree of efficacy can be created in the future.

## 1. Introduction

This paper focuses on the use of inertial sensors (accelerometers and gyroscopes) for the study of movement disorders; specifically, essential tremor (ET) and Parkinson’s disease (PD) are examined. The use of inertial sensors for such a task is quite helpful given the small size and low cost of the MEMS (microelectromechanical systems) inertial sensors that were utilized. Such MEMS inertial sensors are typically smaller than a coin and cost only a few dollars each. This makes them well suited to movement disorders analysis because their small size allows them to be mounted easily in devices used for motion tracking, and their low cost improves accessibility for the use of such devices.

Both ET and PD can have significant negative impacts on the patients that are afflicted with these disorders. Both disorders can affect people’s ability to eat, write and drink, and 73% of ET patients have a documented disability [1]. As well, for PD the estimated annual costs arising from the disorder are approximately $20 billion annually [2]. ET tends to be characterized as an action tremor, meaning that it is present mostly when patients are voluntarily undergoing motion [3] and PD tends to be more of a rest tremor, meaning that it tends to be present when patients are not moving (although it can still be present during motion in many cases) [4]. Both tremors tend to exist in the 3–12 Hz frequency band [5].

Because of the large impact that such disorders can have, it is important to have medications that can adequately treat disorders. This research paper explores methods in which inertial sensors can help to assess the efficacy of treatment options. There have been previous works in which inertial sensors have been used to differentiate movement disorder patient motion from control motion [6,7]; such works have used artificial neural networks with inputs from parameters obtained from inertial sensor data.

Another technique for study of movement disorders involves the use of a Teager Energy Function, which gives an estimate of the overall mechanical energy present during tremor motion [8]. This Teager Energy Function can help researchers to identify periods of significant tremor motion from periods of less substantial tremor motion. The referenced Teager analysis only used accelerometers, and not gyroscopes, and for this reason, the Teager analysis was not able to differentiate between lateral and rotational motion captured in accelerometer signals (as described in Section 2.3 on “Kalman Filtering and Smoothing”). In contrast, the work presented in this paper utilizes a full (six degree-of-freedom) rendering of motion, and this helps to identify (with more specificity) motion associated with the test subjects examined. This in turn makes it easier to identify the patterns of motion associated with ET, PD and movement disorder data.

The work presented also here differs from many previous works primarily in that the techniques discussed can not only help to differentiate control motion from movement disorder patient motion for cases where medication is either not in use of largely ineffective (this kind of analysis has been carried out in many previous works). But the techniques presented here can also be used to help differentiate the motion of controls from effectively medicated PD patients undergoing dyskinesia (which is involuntary motion or the absence of intended motion). This is a significant accomplishment because medicated PD patients often complain about the effects the medication can have on their overall motion, and by helping to quantify such effects, it will be possible to objectively measure the effectiveness of medications, and assist with medical trials to test the efficacy of treatment options proposed. Perhaps it will be possible in the future, using the analysis in this research paper, to develop new treatment options for PD patients, absent the dyskinesia that often accompanies treatment.

## 2. Data Processing Techniques

### 2.1. Continuous Wavelet Analysis

**Figure 1.**
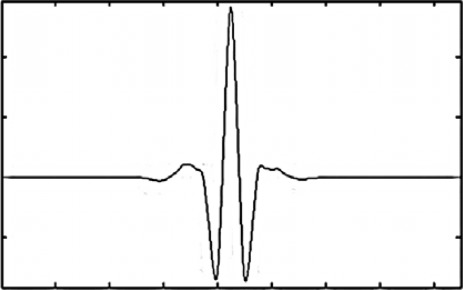
Coiflets 3 mother wavelet.

Continuous wavelet analysis is applied here in part to help to manage frequency spectrum information for the inertial signals under evaluation. Wavelet frequency spectrum data is in many ways similar to a Fourier based frequency spectrum data, but wavelets have a number of inherent advantages when compared to Fourier analysis. For one, there are many families of wavelets from which one can pick and the Coiflets 3 wavelet (the Coiflets 3 mother wavelet is shown in Figure 1) was chosen for analysis because it matched well with the signal under evaluation. In contrast, Fourier analysis tends to be more restricted to sinusoidal based processing techniques when generating frequency spectra.

Another advantage of wavelets is signals sections where data are not good can more easily be removed during analysis (this particular advantage of using wavelets is not particularly useful for the analysis presented here, but it may be useful in future implementations of wavelets in the research area highlighted). Wavelets are well suited to analyzing small signal sections while avoid distortions when processing is taking place near the beginning or end of a signal. This is partly due to the time and frequency resolution that wavelets provide (as opposed to Fourier based analysis which is more focused on only frequency resolution). As a further advantage, if the processing taking place were to utilize the discrete wavelet analysis more broadly for future applications, the wavelet based processing would be much faster than comparable Fourier based processing techniques when generating auto-spectra.

To apply the coiflets 3 mother wavelet and produce wavelet coefficients, *Q*(*m_γ_*, *t_σ_*), the following is used [9]:

(1) $$Q(m_{\gamma },t_{\sigma })=\frac{1}{\sqrt{m_{\gamma }}}\int_{-\infty }^{\infty }s(t)\;\tilde{\psi }\;(\frac{t-t_{\sigma }}{m_{\gamma }})\mathrm{dt}$$

where *m_γ_* is a scaling parameter to appropriately size the wavelet for the analysis, *t_σ_* shifts the wavelet in time so that the wavelet can be applied at the appropriate signal portion, *s*(*t*) is the signal under evaluation as a function of time (*t*) and *ψ̃*(*t*) is the complex conjugate of the mother wavelet *ψ*(*t*) depicted in Figure 1 (this notation for complex conjugate is used throughout this research paper).

What is produced from Equation (1) is a set of values that describe the frequency content of the signal under evaluation in terms of time and frequency evaluated. The frequency under evaluation is not directly available from the wavelet data (which rely on the scaling parameter *m_γ_* to size the wavelets appropriately). However, pseudo-frequency, *F_a_*, at any scale in the wavelet analysis, can be determined by using the following [10]:

(2) $$F_{a}=\frac{F_{c}}{\gamma \Delta t}$$

where the term *F_c_* is the center frequency for the analysis taking place; this term is found by matching a sinusoidal function of known frequency to the mother wavelet shown in Figure 1 such that they overlap as closely as possible. Once the center frequency is known, it can be scaled using the time sampling rate of the data, Δ*t*, and the scaling parameter *γ* (the subscript of *m_γ_* from Equation (1)).

### 2.2. Coherence Analysis

Coherence analysis, as outlined here, is a popular method for biomedical signal evaluation because it allows for comparison of two data streams at frequencies of interest to see how closely two signals match with one another; the coherence analysis outlined here, including the mathematics outlined in this Sub-Section, follows from [11].

Before coherence analysis can be carried out, a signal needs to be split into *L* non-overlapping segments of equal length (where *l* = 1 … *L*). This is done so that statistical validation can later be carried out as given in Equation (6). The number of data points in the non-overlapping signal portions was chosen to be *T* = 128, or approximately one second of data given that the signals were logged at 130 Hz. Given that the data of interest for movement disorders lies in the 3–12 Hz frequency band, segmenting the inertial data into one second intervals was capable of producing quality results in the 3–12 Hz frequency band without being too cumbersome to implement. Cumbersome implementation results if data segments chosen are too long and therefore difficult to extract from experimental data of limited duration in time. Using the above parameters, a discrete signal analysis can be applied as follows:

(3) $$d_{s}\;(\omega ,l)\approx \sum_{k=(l-1)T}^{lT-1}e^{-i\omega k}s(k)$$

where *e* is the exponential function, *i* is an imaginary number and *ω* is angular frequency found from the product of frequency in Hz and 2*π* (where *π* is the ratio of the circumference of a circle to its diameter). The parameter *k* is applied at discrete signal elements for the analysis undertaken. Once Equation (3) has been applied independently to two signals (logged concurrently), the following can be utilized to determine a cross-spectrum [*f̂*_12_(*ω*)]:

(4) $${\hat{f}}_{12}\;(\omega )=\frac{1}{2\pi \mathrm{LT}}\sum_{l=1}^{L}d_{s1}\;(\omega ,l)\;d_{s2}\;\tilde{(\omega ,}l)$$

where *d*_*s*1_(*ω, l*) and *d*_*s*2_(*ω, l*) are found by applying Equation (3) to signals denoted *s*_1_(*k*) and *s*_2_(*k*), respectively. If the cross-spectral term, *f̂*_12_(*ω*), has a large magnitude at a given frequency, it means that the signals evaluated are similar in nature at the frequency evaluated (although they are not necessarily phase matched).

To determine auto-spectra values for a given signal, Equation (4) can be applied to the case where signals *s*_1_(*k*) and *s*_2_(*k*) are the same signal. When signal *s*_2_(*k*) is set to be equivalent to signal *s*_1_(*k*), the auto-spectrum produced is denoted *f̂*_11_(*ω*); and when signal *s*_1_(*k*) is set to be equivalent to signal *s*_2_(*k*), the auto-spectrum produced is denoted *f̂*_22_(*ω*). Using such auto-spectra, the following coherence term can be defined:

(5) $${\left|{\hat{R}}_{12}\;(\omega )\right|}^{2}=\frac{{\left|{\hat{f}}_{12}\;(\omega )\right|}^{2}}{{\hat{f}}_{11}\;(\omega )\;{\hat{f}}_{22}\;(\omega )}$$

where the vertical lines in the numerator and to the left of the equal sign of Equation (5) denote absolute value. The coherence term is defined in such a manner that it scales the cross spectral term to have a value between zero and one. This is useful for comparisons of signals whose magnitudes are inherently not similar.

It is possible to define a 95% confidence limit line (denoted *cl*) for coherence such that any coherence of greater magnitude than this line in a plot can be interpreted as meaningful. Data of lesser magnitude than this line suggests that signals *s*_1_(*k*) and *s*_2_(*k*) are independent. This confidence limit line is found as follows:

(6) $$\mathrm{cl}=1-0.05^{1/(L-1)}$$

Examples exist based on the use of coherence and cross-spectra parameters in comparison of two signal tremors [12,13]. Unfortunately, neither of the two referenced works utilized gyroscopes (they only utilized accelerometers) for data collection. This means that it is not possible to obtain a full (six degree-of-freedom) rendering of motion; in contrast, the work presented here allows for six degree-of-freedom motion tracking (since three accelerometers and three gyroscopes were utilized). With six degree-of-freedom motion tracking, it is easier to identify exactly what the difference in motion is for control, ET and PD data, and this helps to clarify any signal processes analysis undertaken.

### 2.3. Kalman Filtering and Smoothing

Kalman filtering and smoothing are employed for analysis to improve orientation information for signals captured. Such Kalman filtering and smoothing utilize gyroscope data, known start and end orientations for when data is captured and updates from accelerometer data. Start and end orientations are known for the data captured because the IMU (inertial measurement unit) used for signal acquisition is placed in a holster of defined orientation at the beginning and end of each trial. Accelerometer data can be used to provide updates to orientation information when the IMU utilized is not moving significantly; in such cases, accelerometer readings of gravity can be used to calculate IMU orientation for two of the three orientation degrees-of-freedom. Unfortunately, orientation about the gravity vector cannot be captured in such a manner. The scheme outlined here for using Kalman filtering to improve orientation data is described in detail in [14]. There also exists a very similar scheme, as depicted in [15]. After Kalman filtering, Kalman smoothing is applied to the data to improve accuracy, as outlined in [16]. As well, general descriptions of Kalman filtering and smoothing can be found in [17,18].

The reason for using Kalman filtering and smoothing to improve orientation information for signals captured is so that accelerometer data can be corrected to remove gravitational effects. If good quality orientation information is known, then a rotation matrix (*R̂*) can be utilized to transform accelerometer data at each time step into a consistent coordinate frame. Once accelerometer data are in a consistent coordinate frame, gravity can be removed directly from the accelerometer signals before accelerometer data are transformed back into their original coordinate frame; this is outlined in the following equation:

(7) $${\bar{a}}_{t}={\hat{R}}^{-1}(\hat{R}\bar{a}-\bar{g})$$

where the superscript −1 denotes matrix inverse, accelerometer data for the x, y and z (orthogonal) axes are given in respective elements of vector *ā*, gravitational acceleration at a given consistent coordinate frame is given in the three element vector *ḡ* and accelerometer data with gravitational effects removed are given in the three element vector *ā_t_*.

**Figure 2.**
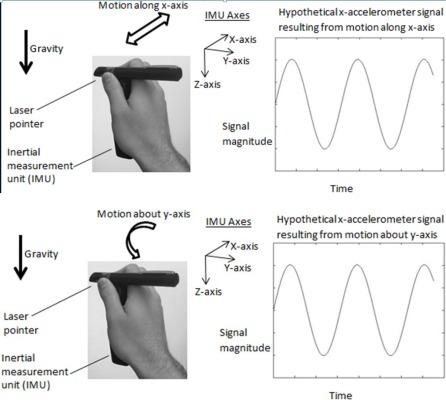
Hypothetical accelerometer signals resulting from lateral and rotational motion.

It may not be intuitively obvious, but raw accelerometer data, which of course contain acceleration from motion tracking and gravitational acceleration, are not suitable directly for analysis of translational tremor. The reason is because rotational tremors also affect accelerometer data as a result of the fact that accelerometers pick up gravitational acceleration. Any rotational tremor about any axis perpendicular to gravity will induce a tremor signal in one or more of the accelerometers logging motion [19]. This phenomenon of an accelerometer signal being affected by rotational motion is depicted in the bottom half of Figure 2; the top half of Figure 2 depicts an x-accelerometer signal resulting from lateral tremor motion along the x-axis. Note that even though the motions depicted in the top and bottom halves of Figure 2 are different from one another (in terms of depicting lateral and rotational motion, respectively), the resulting accelerometer signal created from lateral and rotational motions can be identical (as depicted in the two signals displayed in Figure 2).

For convenience, it is easier to have accelerometer data depicting translational tremor and gyroscope data depicting rotational tremor so that the six degrees-of-freedom analyzed (three translational and three rotational degrees-of-freedom) can be directly matched to data from one of the six inertial sensors used for data capture (there were three accelerometers and three gyroscopes used in the IMU during experimentation). Applying Equation (7) to accelerometer data ensures that what is remaining in terms of tremor motion is largely translational tremor. Gyroscopes, largely will log rotational tremor (and not any other tremors).

For all analysis carried out in this research paper, Equation (7) is applied to all accelerometer data before subsequent analysis. As well, both gyroscope and accelerometer data are filtered to remove much of the frequency information just below 3 Hz so that the 3–12 Hz frequency band of interest for movement disorders can be examined in more detail. The removal of such low frequency data was carried out by zeroing Coiflets 3 discrete wavelet coefficients at a scale *γ* = 2^5^ = 32, which corresponds to a pseudo-frequency of 2.87 (as defined in Equation (2)). All inertial data had low frequency content removed in this manner prior to plotting results. The use of wavelets in this manner is detailed in [20,21].

## 3. Data Collection and Subjects

**Figure 3.**
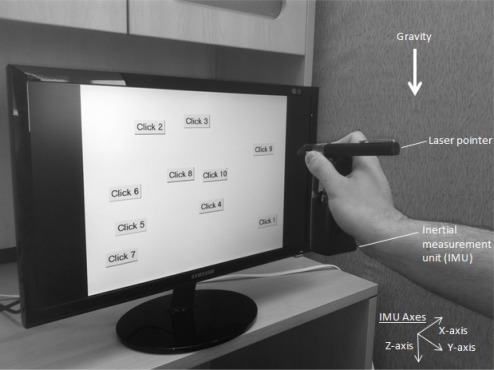
Experimental setup.

Data was captured, as shown in Figure 3, using an IMU containing three accelerometers and three gyroscopes (sensors used were the LIS3LO6AL from ST Microelectronics [22] and the XV-8100CV from Epson Toyocom [23], respectively). Subjects were asked to pick up an IMU out of a holster at the beginning of testing and direct a laser beam from a laser mounted on the IMU at targets of interest on a computer screen; upon arrival at each target, subjects would click a button on the IMU with their thumb before moving onto the next target. Ten such targets of interest were randomly positioned on the computer screen for each trial and ten such trials were carried out with each subject. At the conclusion of testing (for each trial) subjects were asked to place the IMU back in the holster from which they grabbed the IMU at the beginning of the trial. During the course of data collection, subjects were seated. As well, the IMU axes (for both the accelerometer and gyroscope) were defined such that the positive x-axis was pointing to the subject’s right, the positive y-axis was pointing towards the subject when they were holding the IMU in front of them and the positive z-axis was pointing downwards. These orientations were, of course, shifting somewhat during the course of a trial because the IMU orientation was changing as subjects moved the IMU (although, generally, the orientation of the IMU did not change significantly during the course of a trial). The size of the IMU was about the size of the palm of the average test subject, and data was logged on board the IMU without the use of exterior cables.

In total, 11 controls, nine ET and 30 PD patients were utilized. The mean ages for controls, ET and PD patients, respectively, was 64.1, 64.8 and 66.1; and there were four male, six male and 10 male subjects, respectively, for these three groups. The research was approved by the Conjoint Health Research Ethics Board at The University of Calgary and written consent was obtained from each test subject prior to data collection. Medications to reduce movement disorder motion were taken by some of the test subjects, specifically two ET and 27 PD patients were on medication. The ET patients on medication noted that the medication only had a limited impact on their movement disorder motion, but the PD patients sometimes had their movement disorder tremors almost completely mitigated by the medication. In a lot of PD cases, if medication was taken directly before movement disorder data was gathered, tremor motion was much less pronounced. If medication was taken many hours before data was gathered, tremor motion was, in some cases, quite large.

Because the data here presents a mix of non-medicated, slightly medication and heavily medicated subjects, criteria was sought so that movement disorder data could be broken up into groups for subsequent analysis.

Given that the main goal of this paper is to show that both medicated and un-medicated patients can have objective criteria defined to determine the effectiveness of medication and/or the severity of tremor, it was particularly useful to divide patients into groups whereby different motion effects could be examined in a consistent manner within a given group. It was determined that splitting patients into groups of *significant tremor* and *limited tremor* was quite useful to determine the motion present in control and patient data. Wavelets at a scale *γ* = 18 (corresponding to a pseudo-frequency of 5.1 Hz) were found to be quite useful for differentiating between patients with *significant tremor* and patients with *limited tremor*. The peak frequency for movement disorder tremor was often close to 5.1 Hz, which is why wavelet scales corresponding to this frequency were chosen for analysis.

A thresholding criteria was applied to wavelet coefficients (found from Equation (1)) for each test subject. First, the mean for the absolute value of the wavelet coefficients at scale 18 was found for each subject over all trials. The mean for all control data was subsequently found and subjects whose mean was more than one standard deviation above the mean for control data at scale 18 were considered to have *significant tremor*. Subjects whose means’ were not one standard deviation above the mean for control data were found to have *limited tremor*. In order to be classified as having significant tremor, at least one of the six inertial signal means for a subject had to be one standard deviation larger than corresponding mean control data. In total, eight ET patients and nine PD patients were found to have significant tremor. The one ET patient with *limited tremor* is omitted from subsequent analysis because one patient does not generally supply sufficient data so that meaningful analysis can be carried out.

## 4. Results

**Figure 4.**
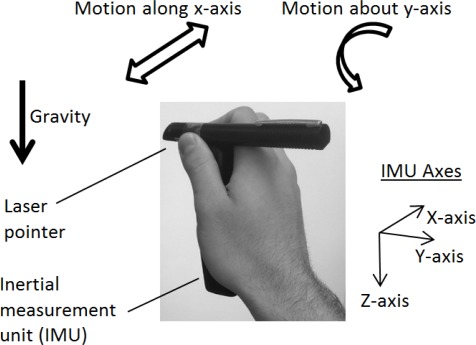
Main concurrent tremor motions (rotational and lateral components) logged.

**Figure 5.**
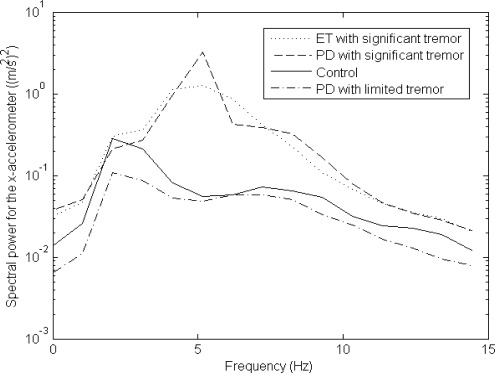
X-accelerometer auto-spectra averaged over the population.

**Figure 6.**
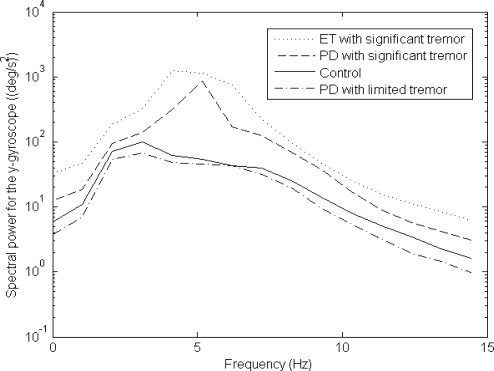
Y-gyroscope auto-spectra averaged over the population.

The principal tremor motion logged was for a subject who had a lateral tremor along the x-axis, as well as (concurrently) a rotational tremor about the y-axis, as depicted in Figure 4. The auto-spectra for the x-accelerometer and y-gyroscope (based on Equation (4)) are given in Figures 5 and 6, respectively. It is clear from the results that there is more frequency content in the 3–12 Hz frequency band for movement disorder patients, and this validates the data collection and processing utilized for the work carried out. Other inertial signals also had similar plots, although the x-accelerometer and y-gyroscope tended to have auto-spectral plots whose magnitude was larger.

The rotational tremor motion about the y-axis was similar to what would be seen if someone was twisting a doorknob back and forth quickly.

**Figure 7.**
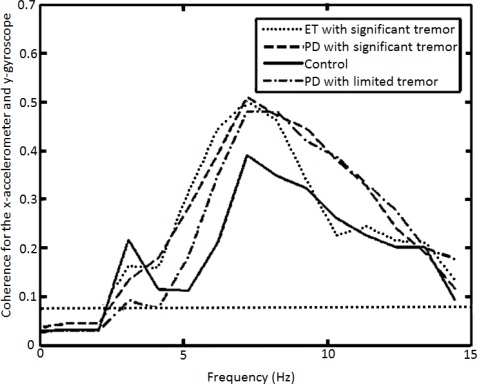
X-accelerometer and y-gyroscope coherence analysis averaged over the populations examined.

Another (less dominant) tremor observed was a lateral z-axis tremor occurring concurrently with a rotational x-axis tremor. This less dominant form of tremor was similar to a clawing motion where someone is striking downwards with his or her hand. A similar pattern to what is seen in Figures 5, 6 and 7 (as explained in the next few pages) is also seen for some subjects when comparing the z-axis lateral tremor and x-axis rotational tremor. Figures 5, 6 and 7 depict the x-axis lateral tremor, which occurred concurrently with the y-axis rotational tremor; the tremor depicted in these figures was present for almost all test subjects (unlike the z-axis lateral tremor, that occurred concurrently with the x-axis rotational tremor). It is because the x-axis lateral tremor and y-axis rotational tremor were so common that they were chosen for analysis. Other than the x-axis lateral and y-axis rotational tremor set, as well as the z-axis lateral and x-axis rotational tremor set, there were no other major tremor types broadly observed from test subjects.

The main significance of the auto-spectral plots, in Figures 5 and 6, is that patients with *significant tremor* can easily have their inertial signals differentiated from control data, given that the *significant tremor* auto-spectra are so much larger in magnitude than the control data auto-spectra in the 3–12 Hz band of interest. However, data for PD patients with *limited tremor* closely match data from controls.

The main purpose of this paper is to show that inertial sensors can be used to help track the efficacy of medications, and in particular to provide a means to objectively quantify dyskinesia in well medicated PD patients. It is clear from Figures 5 and 6 that in cases of large tremor, inertial sensors are quite good at capturing frequency information in the 3–12 Hz band of interest.

However, based on the overall goal of the paper, it is also important to show that in cases of well medicated tremor, inertial sensors are also capable of differentiating patient data from control data. This is essential because many medicated patients report that even though medication may reduce their tremors, it sometimes affects their capacity to move in the manner in which they desire. In other words, PD medication can have some significant side effects (principally dyskinesia) and quantifying this in some way as outlined in this research paper would not only be quite helpful for when testing PD future and current medications and treatment options, but it would also be a completely novel method of identifying and understanding PD movement disorder motion.

Figure 7 outlines a methodology that can be used to differentiate medicated PD patients with *limited tremor* from controls. A useful plot can be produced by applying coherence analysis to the x-accelerometer data (measuring mostly right to left tremor) and the y-gyroscope data (measuring mostly rotational tremor, as if subjects were twisting a door knob back and forth). Other combinations of sensors can also be used to provide similarly useful coherence plots, but the x-accelerometer data and y-gyroscope data were particularly good for this purpose partly because the x-accelerometer logged the most lateral tremor and the y-gyroscope logged the most rotational tremor. Also, these two signals were especially good for coherence analysis because the tremor motion that they logged tended to be of a consistent phase shift [24].

What is particularly interesting about Figure 7 is that all ET and PD data can be differentiated from the data of controls. This is a very significant finding because it allows for PD data with *limited tremor* to be objectively classified as being different from control data.

The horizontal line near the bottom of Figure 7 shows, based on Equation (6), the 95% confidence limit above which coherence should be considered significant. Based on this line, most of the data shown in Figure 7 should be considered significant.

There is an interesting interpretation of the results depicted in Figure 7. Data with *significant tremor* should have more coherence because the x-accelerometer and y-gyroscope chosen for analysis tend to log data for two tremor motions that are quite similar and have a consistent phase shift. On the other hand, PD data with *limited tremor* should not necessarily depict higher coherence than control motion; however, in this case, PD patients with *limited tremor* were almost all medicated and were exhibiting dyskinesia. This higher coherence for PD data with *limited tremor* stems from the fact that controls have more capability to direct the IMU from side to side and up and down whilst only moving along one translational degree of freedom (and not along the other five degrees-of-freedom concurrently). PD patients with *limited tremor*, on the other hand, have less control over there motion (as a result of their dyskinesia) and thus cannot move as easily along only one degree of freedom at a time.

Dyskinesia has not been widely quantified objectively by medical professionals, and generally subjective measures are used to ascertain the severity of dyskinesia based movement (such a giving a patient a score from 1 to 4 to quantify, by eye, the severity of dyskinesia). There is one example of the use of video data to quantify dyskinesia [25], but the use of inertial sensors to quantify dyskinesia (as presented here) is novel. Dyskinesia in Parkinson’s disease is generally a result of the use of drugs used to treat the disease (and, as stated, almost all Parkinson’s patients in this study were taking such drugs). All other major motor symptoms associated with Parkinson’s disease do not explain the results in Figure 7 (making dyskinesia the obvious factor that produced the results given).

Other motions associated with Parkinson’s disease are postural instability, rigidity and bradykinesia (slow movement associated with the disease), as well as tremor. Postural instability is related to inability of patients to keep their balance when walking, which was not examined in this research paper, and therefore could not be a factor for what is depicted in Figure 7. Rigidity and bradykinesia, which result in slow patient motion, by themselves would obviously not directly lead to a higher coherence among tremor motion degrees-of-freedom (similar to what is depicted in Figure 7). Lastly, tremor was ruled out as a factor for what is depicted in Figure 7 for PD patients with *limited tremor* (Figure 5 and 6 verify that such PD patients with *limited tremor* indeed do have small amounts of tremor in their motion). Only dyskinesia, among documented PD motor symptoms, can provide a suitable explanation for what is shown in Figure 7.

To verify that the data given in Figure 7 are significant enough to warrant publication, as statistical analysis was performed using individual peak coherence values for each subject in the 3–12 Hz frequency band. It is important to verify that dyskinesia in PD patients produces statistically different data than control data. Before statistical testing, Q-Q plots (three separate plots) were used to check data sets for normality [26]. The three Q-Q plots used corresponded to the three separate statistical tests in which the three non-control data sets were independently compared to control data sets. Means and standard deviations for the three statistical tests are given in Table 1.

After the data sets were verified to be appropriate for statistical testing, a Welch’s one tailed t-test was applied (Student’s t-test would have been used instead if the population variances were identical) [27,28]. The null hypothesis was that the data sets (three independent tests of two data sets at a time) had the same mean. In each case, it was clear that the data sets were indeed different based on the p-values. The largest p-value from Table 1 was 0.0854, which implies a 8.54% chance of the results observed if the data sets did indeed have the same mean. The results from Table 1 verify what was shown graphically in Figure 7, that the control data can be uniquely identified when compared to the medicated PD patient data.

## 5. Conclusions

The goals of this research paper were to display that inertial data could be used to uniquely identify movement disorder patient data from control data in order to define objective criteria that could be used to quantify the size of tremor for patients and to determine the effectiveness of medication and treatment utilized by patients. More specifically, due to the ability to monitor the effects of dyskinesia, it is possible to evaluate more thoroughly the effects of medication and treatment used by patients; the ability to monitor patient dyskinesia using the methodology depicted in this research paper is novel. For patients with *significant tremor*, it was easy to show that inertial data easily captured large tremor motion. For PD patients with effective medication to reduce tremor, another technique was utilized to show that even though tremor motion was no longer present in patient data, other (dyskinesia based) motion effects could be quantified and used to identify medicated PD patient data when it was been compared to control data. A set of statistical tests verified that all movement disorder patient data presented could be classified as been different from control data. As well, it was explained that PD patients on medications often have difficultly completely controlling there motion (due to dyskinesia), and it is the lack of ability of such patients to move along one translational degree-of-freedom without showing motion for some of the other five degrees-of-freedom that the coherence analysis was able to identify.

Another achievement of this research paper is that Kalman filtering and smoothing were utilized for inertial data pre-processing. This data processing technique was quite useful for the analysis carried out here. Kalman filtering was used to remove rotational motion logged by accelerometers (due to the influence of gravity). After this was done, the three sets of accelerometer data utilized depicted motion along three unique (translational) degrees-of-freedom. This allowed for the coherence analysis in this research paper to be carried out to compare motion along pairs of the six degrees-of-freedom (three translational and three rotational degrees-of-freedom). Most other research conducted using inertial sensors to track movement disorder motion (as referenced in this research paper) does not utilize six degree-of-freedom motion tracking, and so the analysis presented here would not be possible with the data sets presented in these other works.

Other processing carried out included wavelet analysis, which was used to define a threshold to differentiate patients with *significant tremor* from patients with *limited tremor.* Wavelets were also used to define criteria to high pass filter raw inertial data before subsequent processing.

## Figures and Tables

**Table 1.** Results from statistical testing when comparing peak coherence values in the 3–12 Hz frequency band for x-accelerometer and y-gyroscope data.

**Subject Type**	**Mean**	**Standard Deviation**	**P-value using Welch’s one-tailed t-test**
Control	0.480	0.127	-
ET with *significant tremor*	0.660	0.143	0.0067
PD with *significant tremor*	0.650	0.136	0.0055
PD with *limited tremor*	0.548	0.128	0.0854
